# The use of lumbar spine imaging to determine fitness for work in asymptomatic workers who perform manual lifting

**DOI:** 10.47626/1679-4435-2021-882

**Published:** 2024-02-16

**Authors:** Eduardo Myung, Alexander Buarque

**Affiliations:** 1 Associação Paulista de Medicina do Trabalho, São Paulo, SR Brazil

**Keywords:** occupational medicine, occupational health program, low back pain, mass screening, medicina do trabalho, programa de saúde ocupacional, dor lombar, programas de rastreamento

## Abstract

In occupational medicine, evidence-based practices are essential for assessing the accuracy, efficacy, and cost-effectiveness of any technologies used in health programs. This opinion article reflects on the use of imaging tests to screen for workers at risk of low back pain disability and to recommend avoiding tasks that involve high biomechanical risk. The limitations of such testing are discussed through basic epidemiological concepts and evidence collected from systematic reviews.

## Abstract (pt)

A aplicação da saúde baseada em evidências publicadas ou locais na prática da medicina do trabalho é essencial para avaliação da acurácia, efetividade e custo-efetividade de quaisquer tecnologias de saúde implementadas no programa de saúde ocupacional. Neste artigo de opinião, os autores refletem sobre o uso de exames de imagem para detecção precoce de trabalhadores com maior risco de adoecimento por dor lombar associada a inaptidão para atuar em atividades com maior risco ocupacional biomecânico de dorsalgia. As limitações dessa prática são discutidas por meio de conceitos básicos da epidemiologia e evidências publicadas preferencialmente em revisões sistemáticas. Não recomendamos a sua utilização para os objetivos ocupacionais citados neste artigo.

## INTRODUCTION

Routine radiographic screening of the lumbar spine of industrial job applicants began in the 1920s, largely as a way of controlling litigation and compensation costs due to low back pain disability.^1^

Using complementary examinations to determine worker fitness for high-risk activities is a common practice in occupational medicine. As a means of illness and accident prevention, their purpose is to detect changes that indicate a greater risk of disability or risk to the worker and/or coworkers.^2^ As a secondary objective they are used to provide greater accuracy in assessing and documenting worker health status for legal purposes.

The effectiveness, harms, costs, and difficulty of implementing any health technology must be assessed.^3^ Applying health technologies without analyzing published or local evidence increases the risk of harm and ineffective allocation of company resources.

Iatrogenesis, discrimination, and litigation are associated with confusion about screening results, such as the probability that a diagnostic test will be positive when a disease is present and the probability of disease when positive results are found in asymptomatic workers. On the one hand, false positive screening results for low back pain and other musculoskeletal diseases of the spine can result in discrimination and unnecessary treatment and investigation. On the other hand, false negative results can lead to unnecessary litigation.

The subjective and oscillating nature of back pain, in addition to the difficulty of simple, objective, empirical measurement of the biomechanical and psychosocial factors associated with illness in large populations have impeded exposure and outcome measurement in most published studies without resorting to questionnaires.^4^ This has resulted in uncertainty and heterogeneity in the published data and a lack of scientific consensus about the causal links of back pain.

Given that some occupational physicians advocate imaging tests to detect workers who are at greater risk of low back pain disability and, thus, are unable to perform tasks involving greater biomechanical risk, scientific debate about this practice is warranted.

## OBJECTIVES

The purpose of this article is to promote discussion based on scientific evidence, preferably from systematic reviews, about the use of lumbar spine imaging to determine fitness for work in asymptomatic workers who perform manual lifting.

## METHODS

This expert opinion paper is based on manually selected articles from MEDLINE/PubMed. Whenever possible, systematic reviews were prioritized through PubMed search filters. The search was based on the descriptors "Low Back Pain", "Accidents, Occupational", "Diagnosis" and words such as "imaging", "asymptomatic" and "prevention".

## DISCUSSION

Screening, the large-scale organized testing of the general population for early disease detection, is a secondary prevention strategy if associated with preventive interventions. Guiding questions about the usefulness of a specific screening test can be summarized as follows:^3,5^

- Can the disease be detected early?
- What are the test's sensitivity and specificity?
- What is the test's predictive value?
- How serious are the consequences of false positive results?
- What are the monetary, emotional, and resource costs of early detection?
- Does screening promote harm?
- Does early detection lead to health benefits?

Etiological diagnosis of low back pain is often uncertain, and the nature of symptoms often fluctuates, including heterogeneous intensity, frequency, and prognosis. A 2019 systematic review found a lack of objective and accurate etiological diagnostic methods, as well as a low level of scientific evidence in most selected studies.^6^

For the majority of low back pain cases without warning signs, imaging tests are not indicated for etiological diagnosis, since they seldom affect treatment or prognosis.^7,8^

Magnetic resonance imaging will reveal several cases of spinal degeneration in any asymptomatic population. The estimated prevalence of disc degeneration, disc bulge, disc protrusion, and annular fissure in asymptomatic adults aged 20 years is 37%, 30%, 29%, and 19%, respectively, while in asymptomatic adults aged 80 years it is 96%, 84%, 43%, and 29%, respectively.^9^ Although disc degeneration findings are more prevalent in symptomatic than asymptomatic populations,^10^ they cannot be considered predictors of clinical prognosis in a symptomatic population.^11^

The accuracy of magnetic resonance imaging, computed tomography, and myelography for diagnosing disc herniation in a population with low back pain is uncertain. Study limitations, low methodological quality, and heterogeneity of test interpretation criteria contribute to this uncertainty.^12^

Limited scientific evidence suggests that there is an association between the use of imaging tests for low back pain and higher health care costs, greater use of health care services, and increased absenteeism.^13,14,15^ In our experience, imaging examinations unduly reinforce the perception of a causal link between work and disability in symptomatic workers.

There is not enough scientific evidence to affirm that pre-employment screening can prevent musculoskeletal disease by detecting unfit workers. A 2019 systematic review in the Cochrane Database of Systematic Reviews found a small, insufficient, and inconsistent body of evidence with low methodological quality that pre-employment screening could not prevent musculoskeletal disease.^2^ This does not imply there are no benefits to screening, but rather that this topic requires further research.

Evidence is sparse about the efficacy of lumbar spine radiography in pre-employment screening to detect applicants at greater risk of low back pain disability. The findings point to the inaccuracy of prognostic indicators, mistaken perceptions of disability, perceived hiring discrimination due to radiography findings, and unnecessary exposure to ionizing radiation.^1,16,17,18,19^

## CONCLUSIONS

The evidence cited in this article suggests that lumbar spine imaging in pre-employment screening leads to the following problems:

- Limited etiological definition of low back pain (low predictive value in asymptomatic patients)
- Higher pre-employment exam costs with no apparent benefits to employees or employers
- Potential stigmatization of applicants with false-positive results, contributing to presenteeism and absenteeism, as well as unnecessary and/or invasive treatments with uncertain outcomes or treatments that could cause causing additional limitations
- Limitations in defining precise fitness criteria
- Incorrect perceptions among workers about diagnosis, prognosis, causal links, and disability
- Uncertainty about clinical, therapeutic, and occupational conduct in light of test results
- Perception of pre-employment screening as a discriminatory instrument

For these reasons, we do not recommend lumbar spine imaging studies for the occupational purposes mentioned in this paper.
